# Non-pegylated liposomal doxorubicin in older adjuvant early breast cancer patients: cardiac safety analysis and final results of the COLTONE study

**DOI:** 10.1007/s10238-023-01144-8

**Published:** 2023-08-27

**Authors:** Luigi Coltelli, Chiara Finale, Gianna Musettini, Andrea Fontana, Maria Teresa Barletta, Alessandra Renata Lucarini, Iacopo Fabiani, Marco Scalese, Guido Bocci, Luna Chiara Masini, Giulia Soria, Samanta Cupini, Giada Arrighi, Cecilia Barbara, Ermelinda De Maio, Barbara Salvadori, Andrea Marini, Antonio Pellino, Irene Stasi, Michele Emdin, Stefano Giaconi, Lorenzo Marcucci, Giacomo Allegrini

**Affiliations:** 1 Division of Medical Oncology, Leghorn Hospital, Viale Alfieri 36, Leghorn, Italy; 2 Division of Medical Oncology, Pontedera Hospital, Via Roma, 151, Pontedera, Italy; 3 Department of Oncology, Azienda USL Toscana Nord Ovest, Pisa, Italy; 4 Division of Medical Oncology II, Azienda Ospedaliero-Universitaria Pisana, S. Chiara Hospital Via Roma, 67, Pisa, Italy; 5 Department of Cardiology, Azienda Usl Toscana Nord Ovest, Pisa, Italy; 6 Department of Internal Medicine, Azienda USL Toscana Nord Ovest, Pisa, Italy; 7 Cardiology and Cardiovascular Medicine Division, Cardio-Thoracic Department, Fondazione Toscana Gabriele Monasterio, Pisa, Italy; 8 Institute of Clinical Physiology, Italian National Research Council – CNR, Pisa, Italy; 9 Department of Clinical and Experimental Medicine, University of Pisa, Pisa, Italy; 10 Health Science Interdisciplinary Research Center, Scuola Superiore Sant’Anna, Pisa, Italy

**Keywords:** Early breast cancer patients, High risk, Adjuvant chemotherapy, Cardiac safety, Non-pegylated liposomal doxorubicin

## Abstract

**Aims:**

To explore the cardiac safety of adjuvant Non-Pegylated Liposomal Doxorubicin (NPL-DOX) plus Cyclophosphamide (CTX) followed by weekly Paclitaxel, in elderly women (≥ 65 years) with high-risk breast cancer. Previously, we described no symptomatic cardiac events within the first 12 months from starting treatment. We now reported the updated results after a median follow-up 76 months.

**Methods:**

The cardiac activity was evaluated with left ventricular ejection fraction (LVEF) echocardiograms assessments, before starting chemotherapy and every 6 months, until 30 months from baseline, then yearly for at least 5 years.

**Results:**

Forty-seven women were recruited by two Units of Medical Oncology (Ethics Committee authorization CESM-AOUP, 3203/2011; EudraCT identification number: 2010-024067-41**,** for Pisa and Pontedera Hospitals). An episode of grade 3 CHF (NCI-CTCAE, version 3.0) occurred after 18 months the beginning of chemotherapy. The echocardiograms assessments were performed comparing the LVEF values of each patient evaluated at fixed period of time, compared to baseline. We observed a slight changed in terms of mean values at 48, 60, 72 and 84 months. At these time points, a statistically significant reduction of − 3.2%, − 4.6%, − 6.4% and − 7.1%, respectively, was observed. However, LVEF remained above 50% without translation in any relevant clinical signs. No other cardiac significant episodes were reported. To this analysis, in 13 patients (28%) occurred disease relapse and,  of them, 11 (23%) died due to metastatic disease. Eight patients died of cancer-unrelated causes.

**Conclusions:**

The combination including NPL-DOX in elderly patients revealed low rate of cardiac toxic effects. Comparative trials are encouraged.

## Introduction

The treatment of older patients with early breast cancer remains a challenge [1]. Although adjuvant anthracyclines-based chemotherapy has been demonstrated to significantly increase the overall survival of patients regardless age, despite this evidence, in  clinical practice is often not proposed to older patients [2–5]. Thus, the benefit observed in the younger could not be immediately translated in older population. Indeed, the potential efficacy of chemotherapy may be unbalanced by a worsening of comorbidities present in elderly, with the result of a lower tolerability to the treatments in this setting of patients.

Cardiac safety concern, due to an observed higher incidence of cardiovascular toxicity, represents a major challenge to the use of chemotherapies, in particular of anthracyclines, in older patients [6]. Findings from Surveillance, Epidemiology and End Results (SEER) Medicare database showed a significant increased risk of early congestive heart failure (CHF) in patients older than 65 years (hazard ratio 1.26, 95% CI, 1.12–1.4), when treated with anthracyclines-containing regimens at 12 months from the diagnosis, compared to younger populations [7]. Other analyses support the age as a risk factor for CHF when doxorubicin is administered [8]. The increased risk of cardiac toxicities has led to limit the use of anthracyclines in older patients, despite real-world data demonstrate a good toxicity profile in this population to planned anthracycline doses [9].

A new and more manageable therapeutic opportunity in adjuvant setting for the elderly is represented by the liposome-encapsulated formulations [10]. The pegylated (Doxil®, Caelyx®) and the non-pegylated (Myocet®) liposomal doxorubicin compounds have showed the same efficacy but significantly less cardiac, gastrointestinal and hematological toxicity when compared to non-liposomal doxorubicin in metastatic breast cancer patients [11]. Noteworthy, liposome-encapsulated anthracyclines reduce the risk of cardiac toxicity (RR = 0.38, *p* < 0.0001) and the risk of CHF (RR = 0.20, *p* = 0.02) when compared to conventional doxorubicin [12]. Other data confirm the cardiac safety of liposome-encapsulated anthracyclines [13–16], but due to the lack of prospective data in the elderly population, the definitive role of non-pegylated liposomal doxorubicin (NPL-DOX) in this adjuvant setting should still be defined.

Here, we report the updated results of our prospective phase II study, designed and started almost 10 years ago, testing the cardiac safety of an adjuvant regimen including the NPL-DOX combined with cyclophosphamide (CTX), and followed by weekly paclitaxel, in an relatively unselected older patients population with early breast cancer. The first results, published after a median follow-up of 26.5 months, showed no evidence of early episodes of CHF within the first 12 months from the beginning of treatment, the primary end point of the study [17]. After a median follow-up of 76 months, we describe the definitive results in terms of late cardiac safety of this combination. The overall specific cancer survival was also reported.

## Materials and methods

### Patients characteristics, study design and statistical analysis

A full list including characteristics of the patients can be found in the previous publication, to which it refers [17]. Briefly, patients with high-risk early breast cancer requiring adjuvant chemotherapy and aged ≥ 65 years old were enrolled. The study was conducted in two Italian divisions of Medical Oncology: in Pontedera Hospital, Pisa, and in Azienda Ospedaliero-Universitaria Pisana, S. Chiara Hospital, Pisa. Cardiac evaluations were performed at the Cardiology Division of Pontedera Hospital, Pisa, and at Cardiology and Cardiovascular Medicine Division, Cardio-Thoracic Department, Fondazione Toscana Gabriele Monasterio, Pisa, Italy.

A baseline cardiac function assessment was performed, including a baseline left ventricular ejection fraction (LVEF), with a minimum required value at echocardiography > 50%, an electrocardiogram, then followed by a specialist cardiological visit. The electrocardiograms and echocardiograms were repeated at the end of the first three cycles including NPL-DOX, after the last paclitaxel administration or within 6 months from the beginning of chemotherapy and at 12, 18, 24 and 30 months after the study entry, then yearly for at least 5 years.

A multidimensional geriatric assessment (MGA) was performed to exclude frail patients from the study [18]. Based on the responses to MGA questionaries, patients without serious comorbidities and completely independent, or patients with both comorbidities and dependent for some daily activities, entered the study. Complete details of the MGA to exclude frail patients are described in the previous publication [17]. An informed consent was signed. The protocol was approved by the Ethics Committee of the Azienda Ospedaliera-Universitaria Pisana, Pisa, Italy (approval number: CESM-AOUP 3203/2011; EudraCT identification number: 2010-024067-41)**,** for both Pisa and Pontedera Hospitals.

Patients received a sequential schedule of adjuvant chemotherapy including NPL-DOX plus CTX, followed by weekly paclitaxel. The rationale of the choice of this scheme, the doses of chemotherapies, the drugs used to prevent both nausea and vomiting and eventual hypersensitivity reaction have been already reported, as well as the adverse drug reaction assessment and the evaluation of cardiac toxicity [17].

The study was design to test the hypothesis that the NPL-DOX plus CTX combination followed by paclitaxel could determine an early episode of CHF in less than 1% of patients enrolled. The calculated sample size for this single-stage design to assess this reduced incidence of CHF at 12 months was 44 patients with a one-side type I error of 5% and 80% power (H0: *P* <  = P0 vs. H1: *P* >  = P1), according with the single-stage phase II study on the exact binomial distribution proposed by A’Hern [19]. As previously published [17], the statistical analysis of the data was performed with the statistical packages SPSS (13.0), and a *p* value < 0.05 was considered significant. Briefly, the continuous variable LVEF was described both as mean values with standard deviation and median values with 10–90 percentile range. Changes between the LVEF values before the beginning of chemotherapy with the values at 12, 18, 24, 30 months and then yearly were determined by the non-parametric Wilcoxon signed-rank test. Disease free survival (DFS) and overall survival (OS) were secondary end points and estimated from the beginning of the therapy to the date of progression (loco-regional or distant) or to the date of death or if lost at follow up, respectively. Survival probability for DFS and OS was computed using the Kaplan–Meier method.

## Results

### Patients, treatments and general toxicities

Between January 2011 and September 2013, 47 patients with a breast cancer defined at high risk of relapse were enrolled. Baseline characteristics of the patients have been previously reported [17]. Briefly, more than 80% of patients were older than 70 years. Triple-negative cancers were 26%, while HER2-positive tumour at immunochemistry was excluded. Three-quarters of patients had at least a comorbidity.

The treatment was generally well tolerated, with a cumulative total exposure of NPL-DOX plus CTX and of Paclitaxel in the 98% and 94% of patients, respectively. Details of acute toxicities have already been published [17].

### Long-term cardiac effect of the treatment

Compared to previous findings published after a median of 26.5 months [17], here we reported the final analysis of cardiac safety after an almost three-fold longer median follow-up of 76 months (range 68–98), from baseline to the last performed cardiac echocardiogram. As antecedently reported, no episodes of CHF within the first 12 months, the first aim of the study, were observed. In the precedent analysis, we reported an episode of atrial flutter with the complete restoration of heart rhythm after digoxin administration; a mild depression of LVEF (− 10%, − 13%, − 13% and − 15%) in 4 patients (8%), compared to baseline values at 12 months, completely resumed at next visits and a more pronounced decrease in LVEF, from 76% of baseline to 65% at 12th month until to 60% at 24th month, but with no clinical signs or symptoms. A symptomatic grade 3 (scored to NCI-CTCAE, version 3.0 [20]) CHF episode, after 24 months from the baseline, was observed.

The aim of this last analysis was to evaluate the long-term cardiac safety of the combination including NPL-DOX plus CTX, followed by weekly paclitaxel, in terms of LVEF assessments.

Although a quote of patients was not included in the final analysis due to the death of subjects, disease relapsed or lost to follow up, 26, 21, 16 and 15 patients performed a complete cardiac evaluation, respectively, at 48, 60, 72 and 84 months later the first measurement. This allowed us to evaluate the possible late onset of cardiotoxicity due to NPL-DOX plus CTX and paclitaxel in our population. The echocardiograms assessments were performed comparing the LVEF values of each patient evaluated at fixed period of time compared to their baseline assessment. The final analysis revealed a statistically significant reduction in terms of both median and mean values (Table 1 and 2).

**Table 1:** Median (10°-90° quartiles) LVEF (%) value from baseline to 84 months after starting the treatment

Median LVEF (%) value, range *N*° of P.ts	–	60 (58–67) *N* = 46	60 (58–67) *N* = 36	60 (58–65) *N* = 30	60 (56–66) *N* = 26	60 (58–67) *N* = 21	60 (58–65) *N* = 16	60 (58–65) *N* = 15
	**Baseline (B)**	**12 m. from B**	**24 m. from B**	**36 m. from B**	**48 m. from B**	**60 m. from B**	**72 m. from B**	**84 m. from B**
Median baseline LVEF (%) value, range N° of P.ts	60 (56–67) *N* = 47	60 (55–65) *N* = 46	60 (57–65) *N* = 36	60 (545–695) *N* = 30	59 (55–61) *N* = 26	58 (54–64) *N* = 21	59 (54–60) *N* = 16	60 (52–65) *N* = 15
*P*-value* (baseline versus periods of time)	–	0.158	0.673	0.101	0.033	0.015	0.004	0.007

*Calculated with Wilcoxon signed Ranks Test

**Table 2:** Mean LVEF (%) value with standard deviation from baseline to 84 months after starting the treatment

	–	61.4 ± 3.9 *N* = 46	61.5 ± 4.1 *N* = 36	61.2 ± 3.7 *N* = 30	60.8 ± 3.4 *N* = 26	61.5 ± 4.8 *N* = 21	61.4 ± 4.6 *N* = 16	62.1 ± 4.7 *N* = 15
	**Baseline (B)**	**12 m. from B**	**24 m. from B**	**36 m. from B**	**48 m. from B**	**60 m. from B**	**72 m. from B**	**84 m. from B**
Mean LVEF (%) value ± standard deviation	61.4 ± 4.2 *N* = 47	60.5 ± 5 *N* = 46	60.1 ± 4.1 *N* = 36	60.1 ± 5.9 *N* = 30	58.9 ± 4.2 *N* = 26	58.7 ± 4.9 *N* = 21	57.5 ± 3.6 *N* = 16	57.7 ± 4.5 *N* = 15
*P*-value*(baseline vs. periods of time)	–	0.158	0.673	0.101	0.033	0.015	0.004	0.007

*Calculated with Wilcoxon signed Ranks Test

In particular, at these period of time, mean values of -3.2%, -4.6%, -6.4% and -7.1%, respectively, compared to baseline, were observed. Despite the lower values, LVEF remained above 50% and these differences did not translate in any relevant clinical signs, confirming the safety of NPL-DOX in terms of cardiac tolerability profile.

Other common echocardiographic alterations described during the years were aortic sclerosis, left ventricular (LV) hypertrophy, LV diastolic dysfunctions and pulmonary hypertension. These were observed in less than 20% of whole study population, compared to 47 patients enrolled and were generally described as mild alterations and deemed of low clinical relevance, without the need for pharmacological therapy.

### Survival assessment

**Fig. 1:**
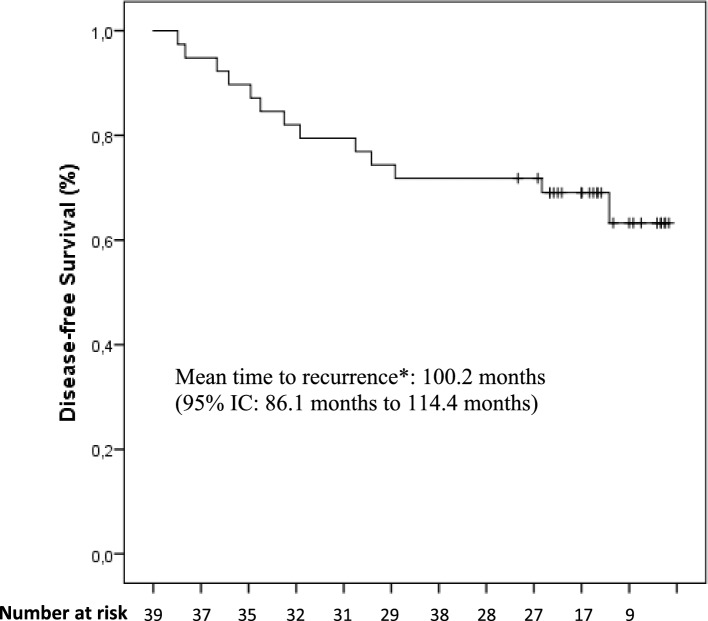
Disease Free Survival curve in patients treated with NPL-DOX, CTX, paclitaxel calculated by the Kaplan–Meier method

**Fig. 2:**
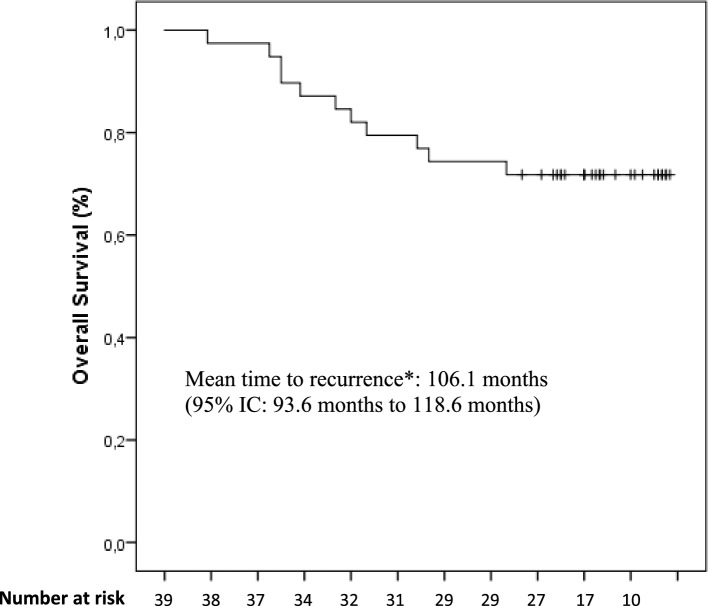
Overall Survival curve in patients treated with NPL-DOX, CTX, paclitaxel calculated by the Kaplan–Meier method

After a median follow-up of 76 months (range, 68–98 months), a total of 13 patients (28%) experienced disease relapse and in 11 of them were observed a distant relapse at the final analysis. Eleven out 13 patients with disease relapse died for progression of disease (23%). Besides these, 8 patients died for causes not related to breast cancer, for a total of 19 deaths (40%). For the patients died without disease relapse, the death occurred in the presence of a previous normal cardiac assessment. To a more careful evaluation of the causes through interviews, the relatives referred sudden death of unknown origin in four patients, a fast decline in cognitive function with deterioration of general condition in two cases and a stroke in two other patients. Ten patients were lost to follow-up after the last control beyond the fifth year. The Kaplan–Meier curve of both DFS and OS is reported in Fig. 1 and 2, respectively. In the analysis of survival, we excluded deaths related to other causes than disease relapse.

## Conclusions

The results of the present study, with a median follow-up of 76 months, confirm the feasibility and the cardiac safety of this adjuvant regimen including NPL-DOX, in an relatively unselected breast cancer elderly population, and fill the void left by the scientific literature on this topic. CHF episodes within the first 12 months from the enrollment, the first end point of the study, were not observed. Compared to our previous report [17], we added LVEF both mean and median values, expression of the cardiac function and closely assessed by repeated echocardiograms, from 36 to 84 months later.

Although the patients evaluated at 48, 60, 72 and 84 months experienced a slight but statistically significant reduction in terms of LVEF values, they remained above 50% over the years. However, these data could lead to speculation a myocardial injury but if so, the damage would have remained sub-clinic, considering that no cardiac clinical signs were observed. Furthermore, due to the characteristics of this elderly population with pre-existing cardiovascular comorbidity, the reduction in mean values could be also attributed to older age besides that to a late effect of doxorubicin-based chemotherapy. Other frequent reported echocardiographic changes were aortic sclerosis, LV hypertrophy, LV diastolic dysfunctions and pulmonary hypertension, but without any appreciable clinical sign.

Overall, regarding the cardiac safety of this combination, only one patient (2%) out of 47 experienced a CHF, 18 months after the start of therapy. This finding should be emphasized if compared with those reported from SEER-Medicare database, with the CHF cumulative incidence of 8% within the first year, when women with age more than 65 were treated with adjuvant anthracyclines [7]. Moreover, just one patient developed an episode of atrial flutter and no other arrhythmias were observed, although they have been described with anthracycline-based treatments, even if uncommon [21]. In addition, no other relevant toxicities were observed. Hematological and non-hematological toxicities experienced by the patients were mild. While details of acute toxicities have been already described [17], no later events were observed, confirming the safety of this combination. Other clinical experiences seem to support the cardiac safety of liposomal anthrcyclines in the adjuvant e neo-adjuvant setting [22–24]. Generally, the cardiac assessment was limited to acute toxicity. Only one study reports a late cardiac evaluation 5 years the start of treatment. In this phase II study, no significant decrease in the median of LVEF was observed when liposomal doxorubicin was administered in elderly patient with breast cancer in neo-adjuvant setting [15, 16].

A possible limitation of our analysis could be represented by the LVEF measurements as the only parameter of cardiac assessment. However, although other approaches have been added over the years, echocardiography still remains today the baseline examination for the evaluation of cardiotoxicity in cancer patients, as reported by the latest guidelines of the European Society of Cardiology [25]. Moreover, when our study was designed, LVEF was the standard test for assessing and monitoring cardiotoxicity.

Over the last years, the efforts have been directed toward identifying early myocardial injury with the intent of starting cardio protective therapy. Echocardiographic left ventricular global longitudinal strain (GLS) represents a new modality to early detect subclinical ventricular dysfunction. The GLS expresses the longitudinal shortening of the myocardial fibers, and it is reported as a negative percentage with respect to the baseline length. Indeed, it provides an accurate assessment of cardiac function and can highlight subclinical abnormalities when traditional echocardiographic parameters are still in a normal range. A meta-analysis and a randomized prospective trial in patients treated with anthracyclines and trastuzumab support the use of the measurement of GLS for the surveillance of cancer therapy-related cardiac dysfunction [26, 27].

In this *scenario*, biomarkers have been introduced in cardio-oncology to predict cardiac toxicity. A recent meta-analysis has evaluated the role of Troponins and of the (N-terminal pro) brain natriuretic peptide (BNP/NT-proBNP). The results have showed that elevated troponins values can predict a left ventricular dysfunction. Therefore, the assessment of troponin levels has been suggested as a test to identify patients who require increased cardiac monitoring or referral to cardio-oncology units, whereas the role of BNP/NT-proBNP remains to define [28]. Finally, the role of troponins to predict cardiotoxicity has been further confirmed in patients treated with anthracyclines and trastuzumab [29].

Based to these findings, in 2022, the European Society of Cardiology suggests adding biomarkers and the measurement of GLS to electrocardiogram and a conventional echocardiogram for monitoring the cardiac activity during cardiotoxic cancer therapy [25].

Pharmacokinetics and pharmacodynamics characteristics of liposome-encapsulated anthracyclines could justify a lower incidence of adverse events compared to conventional doxorubicin. Liposomes hardly cross the vascular endothelium rich in tight capillary junctions, such as the heart muscle and gastrointestinal tract, easily leaving the vascular space when meet a fenestrated endothelium, such as the tumor one, increasing the cancer exposure to doxorubicin [30]. A meta-analysis has compared the safety profile of anthracyclines compared to  liposomal formulations, for the same level of doses, showing significantly less cardiac, gastrointestinal and hematological toxicity [31]. Based on these premises, these updated results support the safety of NPL-DOX, in an relatively unselected older patients with diagnosis of early breast cancer. Although another limitation of our study could be represented by the small number of patients included, the 7-years period of median follow-up represents, to date, the longer time reported in scientific literature, and it supports our conclusion.

Although the lack of comparative studies do not allow us to make definitive conclusion, clinical large experiences seem to confirm also the efficacy of NPL-DOX in early breast cancer. A recent real-world retrospective analysis has evaluated, in more than 1,200 patients, the role of pegylated liposomal doxorubicin (PLD) in adjuvant and neo-adjuvant setting compared to epirubicin. The reported incidence of cardiotoxicity was higher in the epirubicin group than in the PLD group (6.6% vs. 2.2%) with a lower incidence of nausea, vomiting, and myelosuppression, with the same 3-year DFS rate (96.0% vs. 95.1%, *p* = 0.6516) [13]. Moreover, in support of chemotherapy use in elderly, pooled retrospective analysis and results from phase III studies have shown the promising role of adjuvant anthracyclines also in the elderly patients [4–6, 32].

In our study, after a median follow-up of 76 months, a total of 13 patients (28%) experienced disease relapse, confirming the impact on survival of high-risk breast cancer in older patients [33]. Noteworthy, only 8 patients out 47 (17%) died without disease relapse, with a median age of the remaining population in study moved from 70 to 79 years old (range 75 to 84). Overall, all these findings strongly support the necessity to not underestimate the positive role of adjuvant chemotherapy also in this setting of patients. Whereas cardiotoxicity risk remains a concern about adjuvant chemotherapy in elderly population with breast cancer, therapeutic regimens including NPL-DOX could represent a valid alternative to traditional formulations of doxorubicin and epirubicin. Although the small size and the presence of only LVEF measurements, our study has prospectively assessed the role of NPL-DOX, combined with CTX and followed by paclitaxel, confirming its feasibility and long-term cardiac safety. Remarkable, the results seem to support a possible role of NPL-DOX in the elderly, allowing us to treat high-risk older breast cancer patients with an anthracycline-based regimen.
